# Supplementary material for: Projecting XRP price burst by correlation tensor spectra of transaction networks
Source: Sci Rep. 2023 Mar 22;13:4718. doi: 10.1038/s41598-023-31881-5 (PMC10033910; doi:10.1038/s41598-023-31881-5)
Supplement: Supplementary file 1 — Supplementary Information. [file 41598_2023_31881_MOESM1_ESM.pdf]

Supplementary Information: Projecting XRP price burst by correlation tensor spectra of transaction networks

SI Text 1: The daily XRP/USD price

Here we show the daily XRP price between May 18 , 2017 and September 28, 2022 in Fig. S1. It can be observed that the most significant bubble formation and crash of XRP/USD rate occurred at the end of 2017 and beginning of 2018.

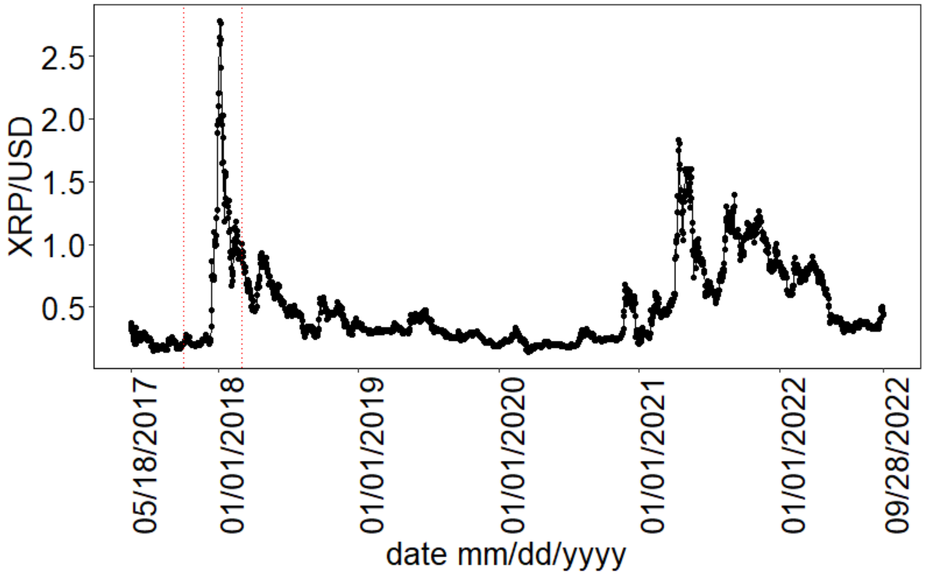

SI Fig. S1. The daily XRP/USD close price (source: https://www.marketwatch.com/investing/cryptocurrency/xrpusd. The region between the red dotted lines indicates the period that we consider for our analysis.

SI Text 2: Dependence of the correlation tensor on window size

The distributions for the elements of the randomized correlation tensors become narrower as the time window $(2\Delta T+1)$ increases, which is shown in Fig. S2 (a). We also show that the largest singular value decreases as the window size increases in Fig. S2 (b). Moreover, the gap between smaller singular values and larger singular values exists for small window sizes, but vanishes as window size becomes large.

SI Text 3: Multilinear regression model for XRP/USD

We perform the following multilinear regression models for the weekly XRP/USD price and report the results in SI Table S1. To calculate the weekly $\overline{\mathrm{XRP/USD}}$ rate, we take 7-day average of the daily close price of XRP/USD using the following expression

$$\overline{\mathrm{XRP/USD}}(t) = \sum_{t'=t-3}^{t+3} \mathrm{XRP/USD}(t')$$

Model 1 : $\overline{\mathrm{XRP/USD}}(t+1) \sim$ intercept + the largest singular value $\rho_1^1(t)$ + the second largest singular value $\rho_2^1(t)$.

The model 1 explains 81% of the variance (adjusted $R^2 = 0.809$) and the significant covariates is only the largest singular value ρ_1^1.

Model 2 : $\overline{\mathrm{XRP/USD}}(t+2) \sim$ intercept + the largest singular value $\rho_1^1(t)$ + the second largest singular value $\rho_2^1(t)$. The model 2 explains 76% of the variance (adjusted $R^2 = 0.758$) and the significant covariates is only the largest singular value ρ_1^1.

Model 3 : $\overline{\mathrm{XRP/USD}}(t+3) \sim$ intercept + the largest singular value $\rho_1^1(t)$ + the second largest singular value $\rho_2^1(t)$. The model 3 explains only 43% of the variance (adjusted $R^2 = 0.433$) and the largest singular value ρ_1^1 is weakly significant.

Model 4 : $\overline{\mathrm{XRP/USD}}(t+3) \sim$ intercept + the largest singular value $\rho_1^1(t)$. The model 4 explains only 47% of the variance (adjusted $R^2 = 0.466$) and the largest singular value ρ_1^1 is significant.

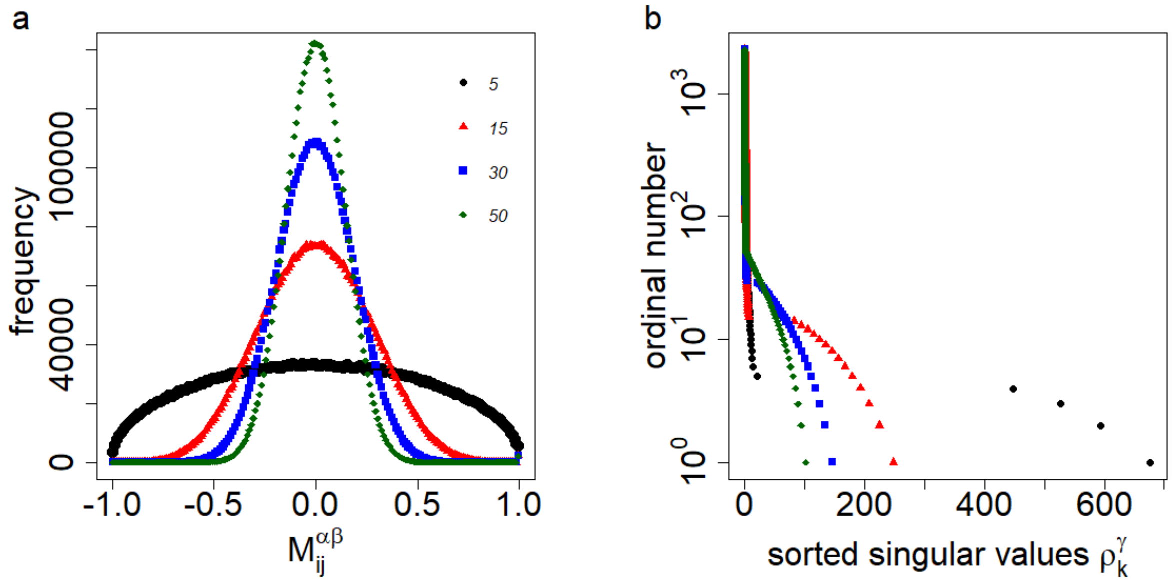

SI Fig. S2. (a) Distributions for the elements of the randomized correlation tensor calculated for different window size. The legends indicate window sizes $(2\Delta T+1)$. (b) Sorted singular values for the randomized correlation tensors.

Table S1. Multilinear regression table for the weekly XRP/USD price.

Variables	Model 1	Model 2	Model 3	Model 4
Intercept	27.914<br>(14.501, 0.073)	36.502 *<br>(15.832, 0.036)	36.796<br>(25.417, 0.170)	28.489**<br>(7.117, 0.001)
The largest singular value, ρ_1^1	-0.033 **<br>(0.0105, 0.006)	-0.038 **<br>(0.011, 0.004)	-0.035<br>(0.018, 0.074)	-0.029 **<br>(0.008, 0.002)
The second largest singular value, ρ_2^1	0.008<br>(0.011, 0.446)	0.0002<br>(0.012, 0.986)	-0.006<br>(0.018, 0.738)	
Observations	18	18	17	17
Adjusted R^2	0.809	0.758	0.433	0.466
p value of F test	1.58×10^{-6}	9.34×10^{-6}	0.007	0.001

The quantities within the brackets represent standard deviations and p-values of the coefficients. We use significance codes for p value: 0 "∗∗∗" 0.001 "∗∗" 0.01 "∗" 0.05 " " 1

SI Text 4: Evolution of regular nodes in the communities of the weekly XRP transaction networks

We use the infomap algorithm to uncover the communities for the weekly networks of XRP transactions. The evolution for the regular nodes of XRP transaction networks within the communities is shown in Fig. S3- S6.

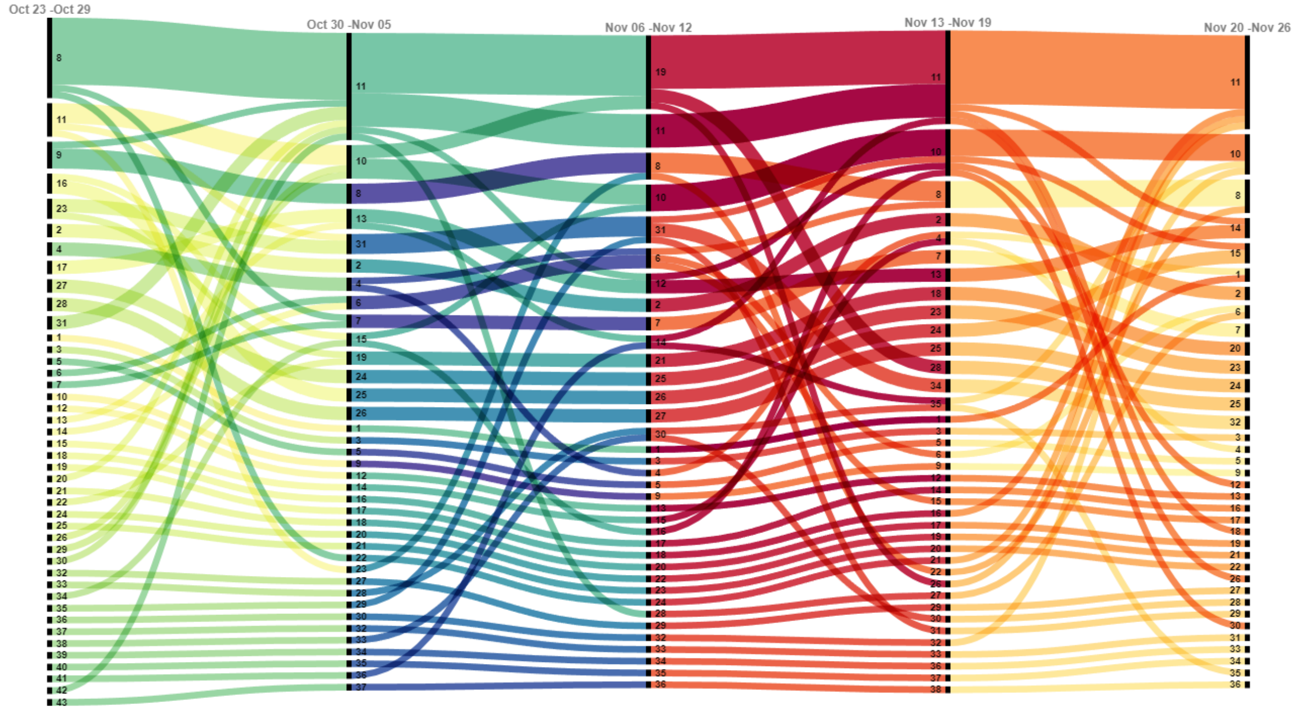

SI Fig. S3. Alluvial diagram for regular nodes showing the evolution of these nodes within the communities of 5 consecutive weekly networks from 2017 October 23 to 2017 November 26. Communities are arranged according to their size and the number indicates the community index.

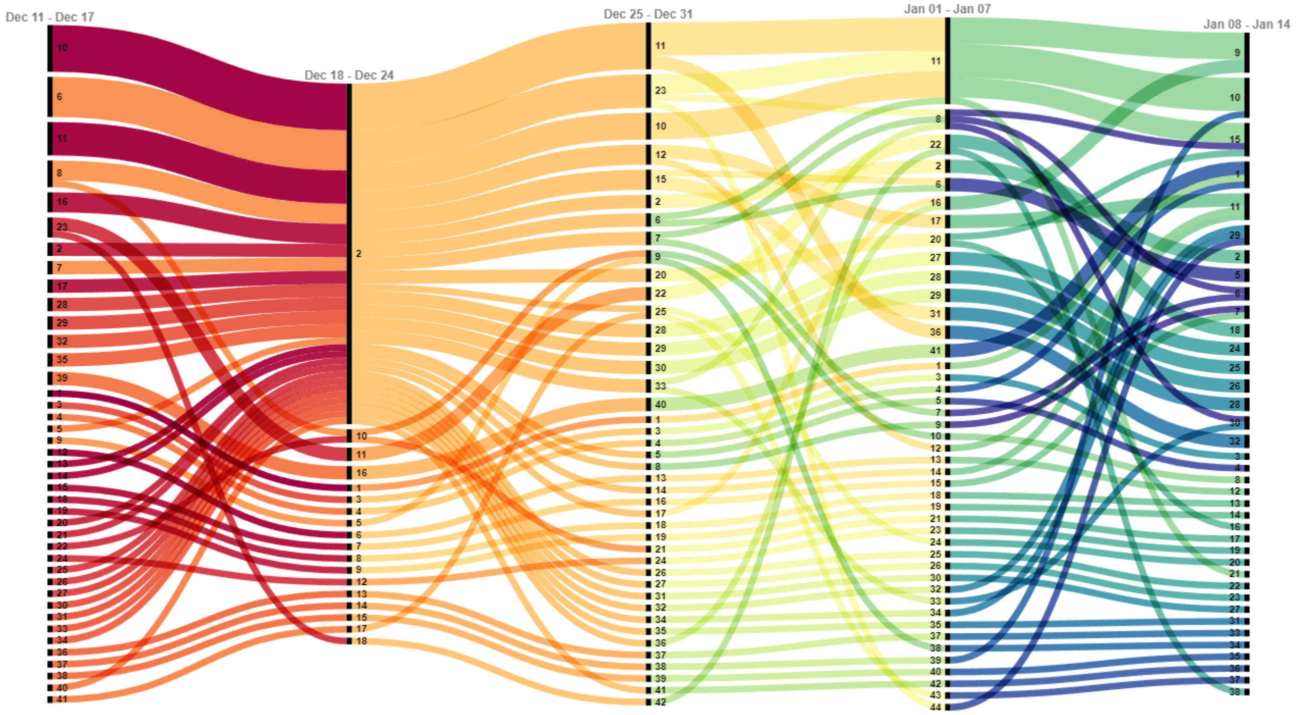

SI Fig. S4. Alluvial diagram for regular nodes showing the evolution of these nodes within the communities of 5 consecutive weekly networks from 2017 December 11 to 2018 January 14. Communities are arranged according to their size and the number indicates the community index.

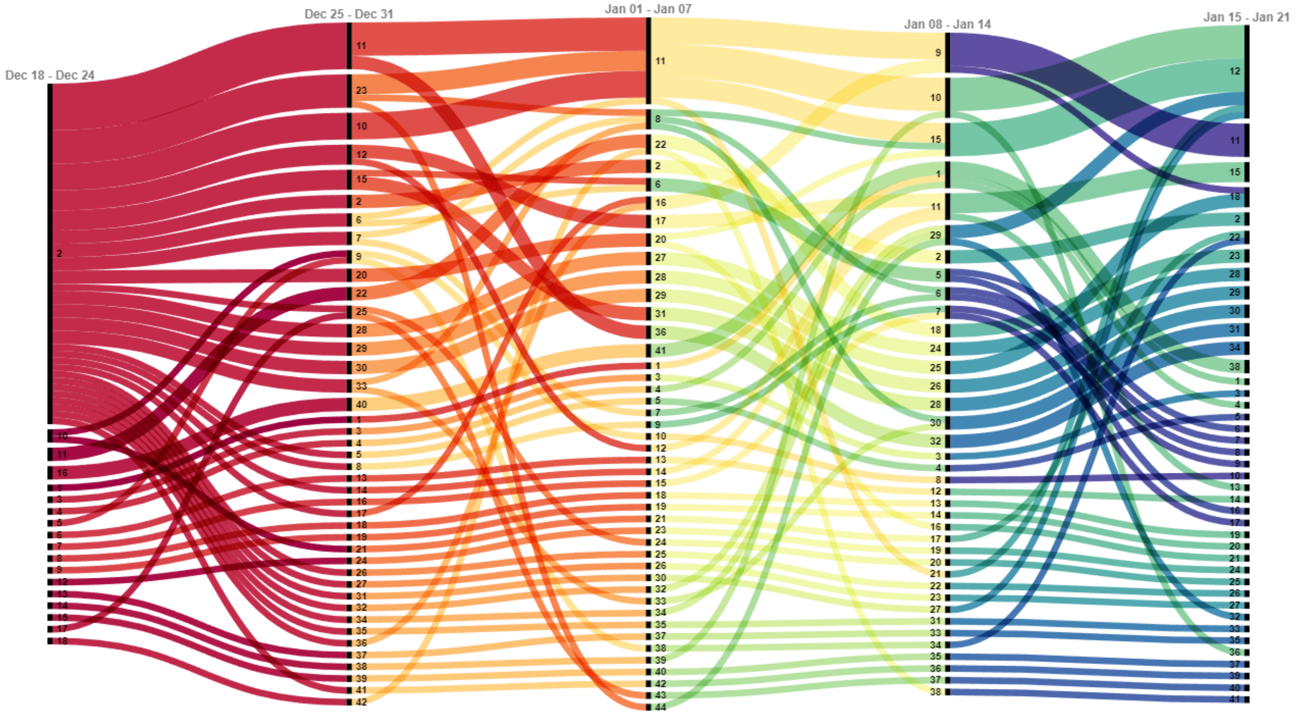

SI Fig. S5. Alluvial diagram for regular nodes showing the evolution of these nodes within the communities of 5 consecutive weekly networks from 2017 December 18 to 2018 January 21. Communities are arranged according to their size and the number indicates the community index.

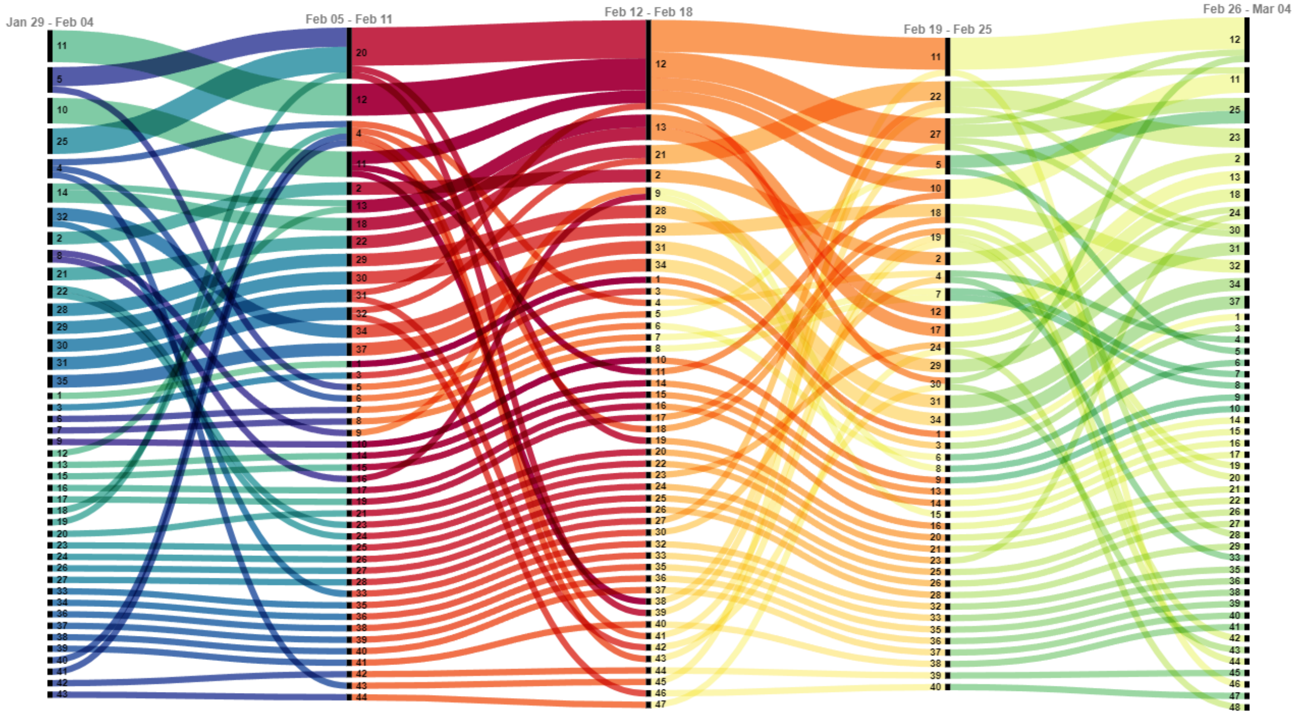

SI Fig. S6. Alluvial diagram for regular nodes showing the evolution of these nodes within the communities of 5 consecutive weekly networks from 2018 January 29 to 2018 March 04. Communities are arranged according to their size and the number indicates the community index.
